# Molecular mechanism of renal lipid accumulation in diabetic kidney disease

**DOI:** 10.1111/jcmm.18364

**Published:** 2024-06-05

**Authors:** Zhengying Fang, Ruijie Liu, Jingyuan Xie, John Cijiang He

**Affiliations:** ^1^ Department of Nephrology, Ruijin Hospital Shanghai Jiao Tong University School of Medicine Shanghai China; ^2^ Barbara T. Murphy Division of Nephrology, Department of Medicine Icahn School of Medicine at Mount Sinai New York New York USA; ^3^ Renal Section James J Peters Veterans Affair Medical Center Bronx New York USA

**Keywords:** cholesterol, diabetic kidney disease, fatty acid, lipid metabolism, oxysterol, SGLT2 inhibitor

## Abstract

Diabetic kidney disease (DKD) is a leading cause of end stage renal disease with unmet clinical demands for treatment. Lipids are essential for cell survival; however, renal cells have limited capability to metabolize overloaded lipids. Dyslipidaemia is common in DKD patients and renal ectopic lipid accumulation is associated with disease progression. Unveiling the molecular mechanism involved in renal lipid regulation is crucial for exploring potential therapeutic targets. In this review, we focused on the mechanism underlying cholesterol, oxysterol and fatty acid metabolism disorder in the context of DKD. Specific regulators of lipid accumulation in different kidney compartment and TREM2 macrophages, a lipid‐related macrophages in DKD, were discussed. The role of sodium‐glucose transporter 2 inhibitors in improving renal lipid accumulation was summarized.

## 1 INTRODUCTION

Diabetic kidney disease (DKD) is a common microvascular complication of diabetes mellitus patients. It is estimated that approximately 40% of individuals with type 2 diabetes and 30% of type 1 diabetes patients had kidney damage due to diabetes.^1^ Regarding the high prevalence of DKD in a growing number of diabetes mellitus patients, DKD has substantially been a leading cause of end stage renal disease worldwide with high disease burden in the past decades.^2^ It has been widely acknowledged that multiple factors are involved in the development and progression of DKD, including metabolic disorders, hemodynamic dysregulation, genetic predisposition, inflammation and fibrosis.^1^ Angiotensin converting enzyme inhibitors, angiotensin receptor blockers, sodium‐glucose transporter 2 (SGLT2) inhibitors, nonsteroidal mineralocorticoid receptor antagonist (MRA) and glucagon‐like peptide 1 (GLP1) receptor agonists are current therapies for DKD management; however, there remained considerable residual risk of progression to renal failure in DKD patients,^3^, ^4^, ^5^, ^6^ indicating an unmet need in targeting pathological events in DKD.

Lipids are crucial biomolecules as they are cellular membrane constituents and function as mediators of various signalling pathways.^7^ Lipids accumulate in the form of lipid droplets, which are organelles that ubiquitously exist in most eukaryotic cells and act an energy pool for membrane synthesis and metabolic demands.^8^ Despite of the essential role of lipids in cell survival, cells in non‐adipose tissue have a limited capacity to deal with overloaded lipids and, therefore, are sensitive to the imbalance of lipid metabolism. Excess cytosolic lipids accumulation in non‐adipose cells resulting in chronic cellular dysfunction and injury are referred as lipotoxicity. Increased subcutaneous abdominal fatty deposits, elevated levels of plasma non‐esterified fatty acids, impaired signalling in adipose tissue and ectopic accumulation of lipids are considered as the main origins of lipotoxicity.^9^

Mounting evidence have shown that a variety of diseases are thought to be promoted by lipotoxicity including chronic kidney disease (CKD).^10^ A prospective study based on a large cohort in Denmark revealed that patients with familial hypercholesterolaemia had a significantly higher risk of CKD after adjusting for multiple clinical parameters, suggesting a central role of lipotoxicity in the development of CKD.^11^ Our recent study demonstrated that non‐alcoholic steatohepatitis is sufficient to induce CKD, providing more direct evidences of the involvement of lipotoxicity in CKD pathogenesis.^12^ As a major cause of CKD, dysregulation of lipid metabolism and lipid deposits have long been described in DKD.^9^ In streptozotocin‐induced diabetic rat, there are marked increases in sterol regulatory element‐binding proteins (SREBPs) and fatty acid synthase (FAS) expression. An in vitro study confirmed that high glucose treatment was able to induce SREBPs and FAS expression, which had a major role in the increased lipid synthesis via do novo lipogenesis and subsequent glomerulosclerosis and proteinuria in DKD.^13^ Similarly, in genetic animal models of type 1 diabetes, it was found that DKD correlated with lipid accumulation, which was resulted from an increase in fatty acid and cholesterol synthesis as well as decreased fatty acid oxidation and cholesterol efflux.^14^

In the following sections, we systemically reviewed the clinical and experimental evidences of the involvement of lipid dysregulation in DKD pathogenesis. Molecular mechanisms underlying glomerular and tubular cholesterol, oxysterol and fatty acid accumulation in DKD were discussed with insights into potential therapeutic targets of renal lipid disorder in DKD. We also summarized the role of SGLT2 inhibitors in regulating kidney lipid metabolism in the context of DKD based on current available reports.

## 2 CHOLESTEROL DISORDER IN DKD

Cellular cholesterol levels are tightly regulated by de novo cholesterol synthesis, cholesterol uptake from bloodstream via low‐density lipoprotein receptor, cholesterol efflux and esterification. SREBP2‐SRECP cleavage‐activating protein (SCAP) complex is an important cholesterol sensor in cellular cholesterol homeostasis. Upon activation by absence of cholesterol, SREBP2 is cleaved and translocates to the nucleus where it promotes transcription of cholesterol synthesis rate‐limiting enzymes. On the contrary, SREBP2‐SCAP complex retains in the endoplasmic reticulum and inhibits cholesterol synthesis when there is sufficient cholesterol in the cell.^15^, ^16^ In addition, excess free cholesterol enhances cholesterol efflux by activating adenosine triphosphate‐binding cassette (ACB) transporter genes and accelerating the esterification of cholesterol by cholesterol acyltransferase (ACAT).^17^

There have been growing evidences that dysregulation of renal cholesterol metabolism contributed to the pathogenesis of DKD.^18^, ^19^, ^20^, ^21^ Farnesoid X receptor (FXR) belonged to the nuclear hormone receptor superfamily and was a key regulator of lipid metabolism.^22^ Lack of FXR accelerated DKD disease progression^23^ while FXR agonist (GW4064 and INT‐747) treatment improved insulin resistance and renal lipid accumulation, as well as ameliorated proteinuria, glomerulosclerosis and tubulointerstitial fibrosis in diabetic mice models by downregulating renal expression of SREBPs.^24^, ^25^ The crucial role of FXR activation in modulation of DKD suggested the importance of cellular cholesterol homeostasis in the DKD pathogenesis.

More importantly, in kidney biopsies of patients with diagnosed DKD, heavy lipid deposition and increased intracellular lipid droplets were observed. The expression of cholesterol uptake receptors was significantly increased, while the cholesterol efflux genes expression was significantly downregulated.^19^ The involvement of cholesterol in the pathogenesis of DKD was supported by the beneficial effects of cholesterol lowing reagents as well. The Fenofibrate Intervention and Event Lowering in Diabetes (FIELD) study showed that fenofibrate, a reagent lowering cholesterol and triglyceride, slowed down the estimated glomerular filtration rate (eGFR) decline and albuminuria progression in DKD patients.^26^ In addition, Simvastatin, a 3‐hydroxy‐3 methylglutaryl coenzyme A (HMG‐CoA) reductase inhibitor lowering LDL‐cholesterol level, was able to improve diabetic albuminuria and reduction of eGFR.^27^

### 2.1 Glomerular endothelial cell cholesterol dysregulation in DKD

Glomerular endothelial cells (GECs) are specialized vascular endothelial cells with fenestrations that form the walls of glomerular tuft capillaries and are the inner layer of the glomerular filtration barrier. GECs are directly exposed to lipoproteins. Oxidized low‐density lipoprotein (Ox‐LDL) has been identified as a product of lipid oxidation, an oxidative stress marker and a potent inducer of foam cells.^28^ Ox‐LDL was able to upregulate intercellular adhesion molecule 1 (ICAM1) expression in GECs by enhancing protein tyrosine kinase (PTK) activity,^29^ and increasing monocyte adhesion to GECs. This effect could be rescued by decreasing PTK activity with its inhibitor or neutralizing ICAM1 by specific antibody.^29^

Adenosine triphosphate‐binding cassette transporter A1 (ABCA1) is a key regulator in cholesterol homeostasis which promotes cholesterol efflux from cells and inhibits inflammatory responses.^30^ ABCA1 expression in GECs was significantly decreased in diabetic apoE^−^/^−^ mice.^31^ High glucose treatment decreased the expression level of ABCA1 in cultured human GECs by downregulating the expression of liver X receptor.^32^ In addition, in human renal GECs cultured under high glucose and high cholesterol conditions, ABCA1 deficiency led to an increase in cellular cholesterol accumulation, endoplasmic reticulum stress and damaged endothelial glycocalyx barrier while overexpression of ABCA1 protected against high glucose and high cholesterol induced glomerular endothelial injury by enhancing cholesterol efflux.^33^ Exendin‐4 is a glucagon‐like peptide‐1 receptor (GLP‐1R) agonist. An in vitro study using human GECs revealed that exendin‐4 decreased renal cholesterol accumulation by up‐regulating the expression of ABCA1, thus increasing cholesterol efflux.^31^

### 2.2 Podocyte cholesterol dysregulation in DKD

Podocytes are terminally differentiated epithelial cells that play an important role in urine filtration, functioning as the outer layer of glomerular barrier. Podocyte loss is one of major pathological characterizers of DKD. Cholesterol homeostasis in podocytes is mainly regulated by de novo cholesterol biosynthesis and cholesterol efflux. An in vitro study showed that there was an increase in cholesterol accumulation as well as actin remodelling when treating cultured podocytes with sera of diabetic patients,^34^ compared to those treated with sera of diabetic individuals without albuminuria. The podocyte cholesterol accumulation in this case was attributed to impaired cholesterol efflux rather than cholesterol biosynthesis, as the expression level of ABCA1, a cholesterol efflux regulatory gene, was decreased by diabetic sera, while the expression of HMG‐CoA reductase, the key enzyme in cholesterol biosynthesis, was not significantly altered.^34^ Furthermore, the induction of cholesterol efflux with cyclodextrin treatment had a renoprotective effect on DKD by decreasing cholesterol accumulation and restoring actin remodelling in podocytes treated with diabetic patients' sera, adding evidence to the importance of cholesterol efflux in DKD podocyte disorders. Another study found that angiotensin II (Ang II) was able to induce cholesterol accumulation in podocytes by downregulating ABCA1 expression, while losartan, an angiotensin receptor blockade, did not prevent cholesterol accumulation induced Ang II.^35^ These studies suggested that cholesterol efflux dysregulation might be a major contributor in DKD podocyte cholesterol accumulation.

Although podocyte specific ABCA1 knockout mice did not show obvious renal injury, podocyte ABCA1 knockout in diabetic ob/ob mice led to higher proteinuria and worsened diabetic kidney injuries compared to wildtype diabetic ob/ob mice,^36^ demonstrating an essential role of ABCA1‐mediated cholesterol efflux in DKD podocytes. Deficiency of ABCA1 in podocytes resulted in accumulation of cardiolipin, which is a phospholipid exclusively located in mitochondria, leading to impaired respiratory chain enzyme acidity and mitochondria dysfunction. Both ABCA1 inducer (A30) and cardiolipin peroxidase inhibitor (E3) were able to reduce cardiolipin oxidation in DKD podocytes, thus preventing progression of the disease.^36^ A recent study focused on drugs that potentially upregulated ABCA1‐dependent cholesterol efflux. By performing phenotypic drug discovery screening assay, the authors found a class of 5‐arylinicotinamide compounds that targeted oxysterol binding protein like 7 and induced ABCA1 expression and cholesterol efflux in cultured podocytes, which might be a potential therapeutic treatment for DKD.^37^ Sterol‐O‐acyltransferase1 (SOAT1) is a cholesterol esterification regulation gene, which converts free cholesterol to cholesterol esters at the endoplasmic reticulum. Interestingly, similar to ABCA1 knockout mice, SOAT1 knockout mice did not develop proteinuria or significant diabetic kidney injury. However, SOAT deletion or inhibition in experimental model of DKD led to reduced cholesterol ester content in the kidney cortices and indeed protected DKD mice from the disease progression.^38^ This indicated that free cholesterol accumulation might be an accelerator rather than a causal factor in the pathogenesis of DKD. G‐protein‐coupled receptor 43 (GPR43) is a posttranscriptional regulator that involved in cholesterol metabolism. GPR43 activation resulted in increased cholesterol influx and autophagy inhibition in podocytes. In vivo knockout of GPR43 alleviated cholesterol accumulation in diabetic mice kidneys. Both genetic deletion and pharmacological inhibition of GPR43 prevented cholesterol accumulation in high glucose‐stimulated podocytes.^39^

### 2.3 Tubular cell cholesterol dysregulation in DKD

Lipid accumulation in tubular cells is common in DKD patients.^19^ Multiple studies have reported that ectopic renal lipid accumulation was positively correlated with tubulointerstitial injuries in DKD.^21^, ^40^ An in vitro study conducted in rat renal tubular epithelial cells found that high glucose treatment upregulated SREBPs and their targeted genes FAS and HMG‐CoA reductases, causing cellular lipid accumulation.^41^ Recent studies have further revealed cellular cholesterol homeostasis regulators, which played a crucial role in DKD tubule injuries.

Disulfide‐bond A oxidoreductase‐like protein (DsbA‐L) is a member of the glutathione S‐transferase superfamily, which has a wide variety of biological functions including regulating lipid metabolism.^42^, ^43^ Other than adipose tissues, DsbA‐L was found to be abundantly expressed in renal proximal tubular cells.^44^ It was demonstrated that by regulating the expression of HMG‐CoA reductase, a rate‐limiting enzyme for cholesterol synthesis, through phosphorylation of adenosine monophosphate kinase, DsbA‐L conquered a protective role in lipid‐related renal injuries in both DKD mice as well as DKD patients.^45^ Phosphofurin acidic cluster sorting protein 2 (PACS‐2), a member of PACS family, was involved in the lipid metabolism by regulating the formation of endoplasmic reticulum lipid‐synthesizing centres at the mitochondria‐associated endoplasmic reticulum membranes (MAM).^44^ Reduction of PACS‐2 via adenoviral shRNA delivery improved mitochondrial oxidative capacity and glucose metabolism in ob/ob mice.^46^ In kidney samples of DKD patients, PACS‐2 was predominantly expressed in the renal tubules. Of note, PACS‐2 expression level was positively correlated with renal function and negatively correlated with tubulointerstitial injuries degrees in DKD patients. Conditional deletion of Pacs‐2 in proximal tubules led to worsened tubular lesions in diabetic mice models, accompanied with pronounced changes in MAM formation and defective mitophagy.^47^ Additionally, in high fat diet plus streptozotocin‐induced diabetic mice, tubule‐specific deletion of Pacs‐2 upregulated the expression of SOAT1, followed by the activation of the downstream SREBPs, thus inhibiting cellular cholesterol efflux and promoting renal lipid accumulation.^48^

## 3 OXYSTEROL DISORDER IN DKD

Oxysterols are a group of oxidized forms of cholesterol or of its precursors, which arise from dietary sources and enzymatic oxidation as well as non‐enzymatic oxidation reactions both in vivo and ex vivo. They have additional oxygen functions as hydroxyl, carbonyl or epoxide groups.^49^ Dietary oxysterols include 7‐ketocholesterol (7KC), 7α‐hydroxycholesterol, 7β‐hydroxycholesterol and α‐ and β‐5,6‐epoxycholesterol. Oxysterols derived by specific enzymatic reactions are formed mostly by cytochrome P450 (CYP) enzymes, including 24(S)‐hydroxycholesterol formed by CYP46A1, 7α‐hydroxycholesterol formed by CYP7A1 and 27‐hydroxycholesterol formed by CYP27A1. Production of enzymatically produced oxysterols is regulated by enzymes at their protein or activity levels, while levels of non‐enzymatically produced oxysterols are regulated by oxidant and antioxidant agents. Some oxysterols functioned as ligands for receptors and have been shown to be important regulators of cholesterol homeostasis.^50^, ^51^

**FIGURE 1:**
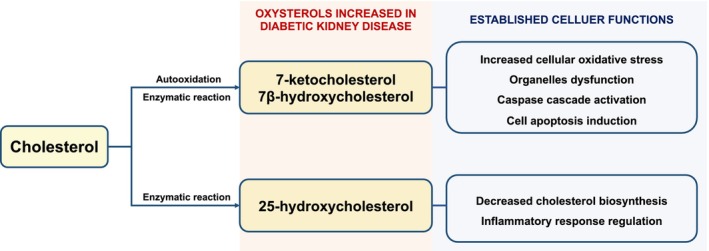
7‐ketocholesterol, 7β‐hydroxycholesterol and 25‐hydroxycholesterol are oxysterols that increased in diabetic kidneys. These oxysterols are known to have multiple effects on cellular function and survival. Further studies are needed to elucidate their role in diabetic kidney disease.

Oxysterols are more hydrophilic than cholesterol, which makes them easier to cross hydrophilic barriers, including the glomerular filtration barrier. By utilizing gas chromatograph mass spectrometry, Kumar et al. successfully detected oxysterols in urine samples and found a variety kinds of oxysterols in rats urine.^52^ In plasma, oxysterols levels were maintained at relatively low concentrations, especially when compared to plasma cholesterol levels (approximately 10^−3^–10^−6^ fold).^53^ Interestingly, multiple studies have shown that in both diabetic patients and diabetic animal models, plasma oxysterols levels were significantly higher in diabetic individuals than in healthy controls^54^, ^55^, ^56^, ^57^, ^58^, ^59^ (Figure 1). Yoshioka et al. showed that plasma levels of 7KC, 7α‐hydroxycholesterol and 7β‐hydroxycholesterol were significantly upregulated in streptozotocin‐induced diabetic rats kidneys than in control rats kidneys.^60^ It is known that 7KC and 7β‐hydroxycholesterol can trigger cellular oxidative stress and cause multiple organelles injury, particularly mitochondrial and peroxisomal dysfunction, leading to cell death.^61^ Another oxysterol of interest in DKD is 25‐hydroxycholesterol (25HC), which is generated from cholesterol via enzymatic reaction by cholesterol‐25 hydroxylase (CH25H). It was shown that diabetic patients had higher plasma 25HC levels than healthy controls^54^ despite of its relatively low absolute plasma concentration compared to other plasma oxysterols. Another study revealed that CH25H ranked as one of the top upregulated genes in glomerular endothelial cells of diabetic db/db mice, compared to non‐diabetic control mice glomerular endothelial cells.^62^ 25HC dysregulation has been linked to several metabolic and vascular diseases.^63^, ^64^, ^65^, ^66^ It was found that 25HC mediated SREBP transcriptional activity, involving in the cell lipid regulation.^67^, ^68^ Besides, 25HC was also found to be highly expressed in immune cells and could regulate inflammatory response under disease conditions.^63^, ^64^ Further studies are required to explore the role of 25HC in the context of DKD. The development of the new mass spectrometry imaging technique enables the researchers to image cholesterol and its derivatives including oxysterol in animal models and from human biopsy samples, providing a new method to help better study the cholesterol and oxysterol metabolism in diseases.^69^

## 4 FATTY ACID DISORDER IN DKD

As an essential cellular energy source, free fatty acids are converted to fatty acyl‐CoA by acyl‐CoA synthetase before entering mitochondrion with the help of carnitine palmitoyltransferase1. By β‐oxidation, mitochondrial fatty acyl‐CoA was further converted to acetyl‐CoA, which is essential for adenosine triphosphate (ATP) production. Overloaded acetyl‐CoA exceeding cellular energy demands are converted back to fatty acid and are stored in the form of triglyceride. Fatty acid accumulation was observed in diabetic kidneys and was found to be resulted from accelerated fatty acid synthesis and decreased fatty acid oxidation.^70^ Several regulatory genes were involved in the fatty acid homeostasis, including SREBPs and carbohydrate‐response element‐binding protein (ChREBP) which activate fatty acid synthesis, peroxisome proliferator‐activated receptor alpha (PPARA) and peroxisome proliferator‐activated receptor‐γ coactivator (PGC)‐1α, which regulate fatty acid oxidation. It was reported that diabetic kidneys had increased levels of SREBP and ChREBP and decreased levels of PPARa and PGC‐1α, leading to fatty acid accumulation.^70^

The acetyl‐CoA carboxylase‐encoding genes ACACA and ACACB encode acetyl‐coenzyme A carboxylase α (ACCα) and acetyl‐CoA carboxylase β (ACCβ), which are key rate‐limiting enzymes of β‐oxidation of fatty acid.^71^ A longitudinal study including over 90 American Indians with type 2 diabetes showed that higher serum unsaturated free fatty acids were positively correlated with DKD progression. DKD progressors had significantly higher relative abundance of polyunsaturated triacylglycerols (TAGs) and a lower abundance of C16‐C20 acylcarnitines (ACs), where renal expression of ACACA correlated with the lipidomic changes.^72^ Case–control studies have shown a significant association between a single nucleotide polymorphism rs2268388 located in ACACB gene and type 2 diabetes‐related nephropathy (T2DN),^73^, ^74^ where the T allele was a risk factor of T2DN development.^75^

### 4.1 Podocyte fatty acid dysregulation in DKD

Research has shown that podocyte fatty acid accumulation was positively correlated to albuminuria.^76^ Free fatty acid‐bound albumin, rather than free fatty acid‐free albumin, was able to induce podocyte fluid‐phase uptake in the form of macropinocytosis.^76^ Macropinocytosis is a form of endocytosis of some specific cell types to ingest extracellular liquid and dissolved materials, such as solute molecules, nutrients and antigens. Free fatty acid related macropinocytosis in podocytes was triggered by free fatty acid binding to lipid binding G‐protein‐coupled receptors (GPCRs), including free fatty acid receptors1, 2 and 3 (FFAR1, 2, 3), followed by activation of the Gβ/Gγ complex and RAC1, a member of Rho GTPase family and an important factor for podocyte cytoskeleton organization.^76^ A recent study found that PBI‐4050, an FFAR1 agonist, conquered a renoprotective effect in eNOS^−^/^−^ db/db diabetic mice kidneys.^77^ Fatty acid translocase (FAT) (also called as CD36) is a transmembrane protein that mediates cellular fatty acid uptake. The expression of CD36 was increased in kidney tissue of DKD patients with hyperlipidaemia.^78^ An in vitro study conducted in conditionally immortalized mouse podocytes showed that specific inhibitors of the fatty acid binding site on FAT/CD36 attenuated palmitic acid induced fatty acid accumulation, reactive oxygen species production and podocyte apoptosis,^78^ suggesting the involvement of FAT/CD36 in diabetic podocyte fatty acid disorders. Junctional adhesion molecules (JAMs) are members of immunoglobulin superfamily proteins which expressed at cell junctions in epithelial cells and endothelial cells as well as in leukocytes, platelets and erythrocytes.^79^ Aside from various biological functions (e.g. cell polarity regulation, epithelial barrier formation, cell migration regulation), JAM family members also had a role in lipid metabolism and dyslipidaemia associated disease.^80^ Intriguingly, one of the JAMs, junctional adhesion molecule‐like protein (JAML), had an elevated level in the glomeruli of diabetic mice models and in patients with DKD compared to control subjects.^81^ More importantly, the expression level of JAML was positively correlated with renal lipid accumulation and negatively associated with the glomerular filtration rate in DKD individuals. JAML deficiency ameliorated renal lipid accumulation by downregulating SREBP1 expression through the sirtuin1‐mediated AMP‐activated protein kinase signalling pathway.^81^

### 4.2 Tubular epithelial cells fatty acid dysregulation in DKD

Tubular epithelial cells, especially proximal tubular cells (PTC), have high levels of baseline energy consumption and use fatty acid as preferred energy substrate for energy generation.^82^ Therefore, tubular epithelial cells are more susceptible to fatty acid metabolism disorders. In the non‐diseased condition, fatty acid enters the renal proximal tubule cells bidirectionally via FAT/CD36 located at the basolateral membrane as well as by endocytosis at the apical membrane side in an albumin fatty acid‐bound form.^83^, ^84^ Proximal tubular cells hydrolyse the albumin‐bound fatty acids into free fatty acid and store them in the energy pool inside the cells.^70^ In DKD with urinary albumin loss, fatty acid‐bound albumin was massively taken up by proximal tubule cells.^85^, ^86^ Elevated free fatty acid generated cellular mitochondrial dysfunctions, which led to incomplete FAO and excess production of reactive oxygen species,^87^ aggravating tubulointerstitial injuries.

Fatty acid transport proteins (FATPs) are highly conserved proteins that involve in fatty acid uptake by facilitating transport of fatty acid across the plasma membrane.^88^ FATP2, the protein encoded by the Slc27a2 gene, was identified as the most abundant FATP in kidney^89^, ^90^ and was predominately expressed in apical proximal tubule cells.^91^ Khan et al. generated Lepr^db/db^ eNOS^−/−^ mice with global Slc27a2 deletion and induced the mice with high fat diet plus low‐dose streptozotocin to mimic obesity, type 2 diabetes and DKD. It was demonstrated that FATP2 deletion resulted in improvements in DKD histopathology and renal function.^92^

Kidney injury molecule (KIM)‐1 is a key mediator in PTC uptake of palmitic acid‐bound albumin, which led to PTC mitochondrial fragmentation, DNA damage response and cell death, resulting in renal interstitial inflammation.^93^ It was reported that KIM‐1 expression level was elevated in diabetic patients and mice kidneys. Moreover, in diabetic mice, both genetic deletion of functional domain of KIM‐1 as well as pharmaceutical inhibition of KIM‐1 attenuated DKD kidney injury, by mediating palmitic acid‐bound albumin uptake; this indicated a potential therapeutic role of KIM‐1 inhibitors in DKD via regulating PTC fatty acid uptake.^93^

## 5 LIPID METABOLITES IN DKD

Emerging studies have also shown the involvement of lipid metabolites including phospholipids and adiponectin in the regulation of DKD. Phosphatidylethanolamine (PE) is one of the most abundant phospholipids in eukaryotic cells, whose roles in neurodegenerative and metabolic diseases have invoked great interest.^94^ It has been shown that injection of PE led to DKD‐like phenotypes in zebrafish larvae including pronephros alterations and glomerular basement membrane thickening, suggesting its critical role in DKD development.^95^ Urinary levels of lysophosphatidylcholine (LPC), another essential phospholipid, were found to be associated with rapid progression of DKD in a prospective cohort. Further studies in spontaneously diabetic Torii fatty rats revealed that increased urinary LPC was derived from the kidney other than the blood stream, and ectopically accumulated LPC in kidney tissue was associated with DKD progression.^96^ An in vitro study confirmed that LPC led to accumulation of lipid droplets, cell lipotoxicity and death in cultured proximal tubular cells.^96^ Together, these studies highlighted the critical roles of phospholipids in DKD.

Adiponectin is an adipokine that predominantly secreted by adipose tissue and has pleiotropic functions in multiple tissues including kidney.^97^ Clinical studies have reported that urinary levels of high molecular weight adiponectin correlated with eGFR decline in DKD patients.^98^, ^99^ Genetic ablation of adiponectin in mice led to podocyte foot processes fusion and albuminuria which were partially rescued by adiponectin supplement, suggesting a protective role of adiponectin in renal injuries.^100^ A recent study revealed that adiponectin had a direct effect in glomerular endothelial cells. Intraperitoneal injection of globular adiponectin restored glomerular endothelial glycocalyx depth and reduced albumin permeability in the glomeruli of db/db mice.^101^

## 6 LIPID‐RELATED MACROPHAGE IN DKD

Accumulating evidence have suggested that lipid dysregulation in kidney macrophages contribute to renal injury in chronic kidney disease.^102^, ^103^ Triggering receptor expressed on myeloid cells 2 (Trem2) high expressing macrophage is a subset of lipid‐associated macrophage which was first described in adipose tissue immune cells.^104^ It is known that Trem2 signalling played a vital role in the macrophages in response to disrupted lipid homeostasis in metabolic diseases.^105^, ^106^ Genetic global ablation of Trem2 in mice led to systemic hypercholesterolaemia, body fat accumulation and glucose intolerance.^104^ By utilizing single cell sequencing technique, recent studies identified that Trem2 high expressing macrophage significantly expanded in OVE26 diabetic mice as well as high fat diet fed BTBR ob/ob diabetic mice.^106^, ^107^ Moreover, the increase in the Trem2^+^ infiltrating macrophage was validated in human DKD renal biopsy samples and were observed in both glomerular and tubulointerstitial areas.^107^ It is worth further exploring the effect of the Trem2 high expressing macrophage on renal lipid metabolism and its role in DKD lipid dysregulation in future studies.

## 7 THE ROLE OF SODIUM‐GLUCOSE TRANSPORTER 2 INHIBITOR IN RENAL LIPID METABOLISM

SGLT2 is a renal low‐affinity high‐capacity sodium‐glucose cotransporter, which predominantly expressed at the apical membrane of S1 and S2 segments of the renal proximal convoluted tubules.^108^, ^109^, ^110^ Under physical condition, over 90% of filtered glucose is reabsorbed by SGLT2.^111^, ^112^ Loss of function mutations in SGLT2 coding gene SLC5A2 has been known to lead to familial renal glycosuria, without affecting the lifespan of the patients.^113^ Therefore, the SGLT2 inhibitors has been developed as blood glucose regulators.^114^ Multiple randomized controlled trials have demonstrated the ability of SGLT2 inhibitors to improve composite renal outcomes in patients with type 2 diabetes mellitus.^115^, ^116^, ^117^, ^118^ Of note, systematic review and meta‐analysis showed the efficacy of SGLT2 inhibitor in slowing the progression to end stage kidney disease in patients with established type 2 diabetes nephropathy, and the beneficial effect is consistent across studies.^119^ However, regardless of the approval and use of SGLT2 inhibitors in managing patients with DKD, the underlying mechanism of the renal protective role of SGLT2 inhibitors remains largely unclear.

Several studies have explored the potential mechanisms of the effect of SGLT2 inhibition on preventing DKD from disease progression in the aspect of renal lipid regulation. It was found that SGLT2 inhibitor treatment decreased cholesterol level^120^ and prevented renal lipid accumulation by decreasing key transcriptional factors and enzymes involved in fatty acid and triglyceride synthesis, including carbohydrate‐responsive element‐binding protein‐β, pyruvate kinase L, SCD‐1 and DGAT1.^121^ A more recent study further showed that the SGLT2 inhibitor canagliflozin enhanced renal fatty acid oxidation with increased ATP contents and carnitine palmityl transferase‐1A expression, resulting in ameliorated renal lipid droplet accumulation in type 2 diabetic db/db mice.^122^ Interestingly, research also found that SGLT2 inhibitor dapagliflozin treatment in db/db diabetic mice altered the concentration of membranous lipids in the kidney cortex based on mass spectrometry lipidomics.^123^ The role of SGLT2 inhibitors in renal lipid regulation in the context of DKD was also supported by human study. A moderate size of prospective study demonstrated that diabetic patients treated with metformin and empagliflozin had lower advanced glycation end products levels and renal fat accumulation than those treated only with metformin.^120^

Taken together, these data indicated a critical role of SGLT2 inhibitors in regulation of lipid metabolism in DKD. However, more studies are needed to confirm these findings. It is unclear whether the beneficial role of SGLT2 inhibitors in mediating renal lipid metabolism is a direct drug effect or is secondary to the systemic glucose metabolism improvement. Besides, it is also unknown whether the effect is consistent among all classes of SGLT2 inhibitors.

## 8 CONCLUSION

**FIGURE 2:**
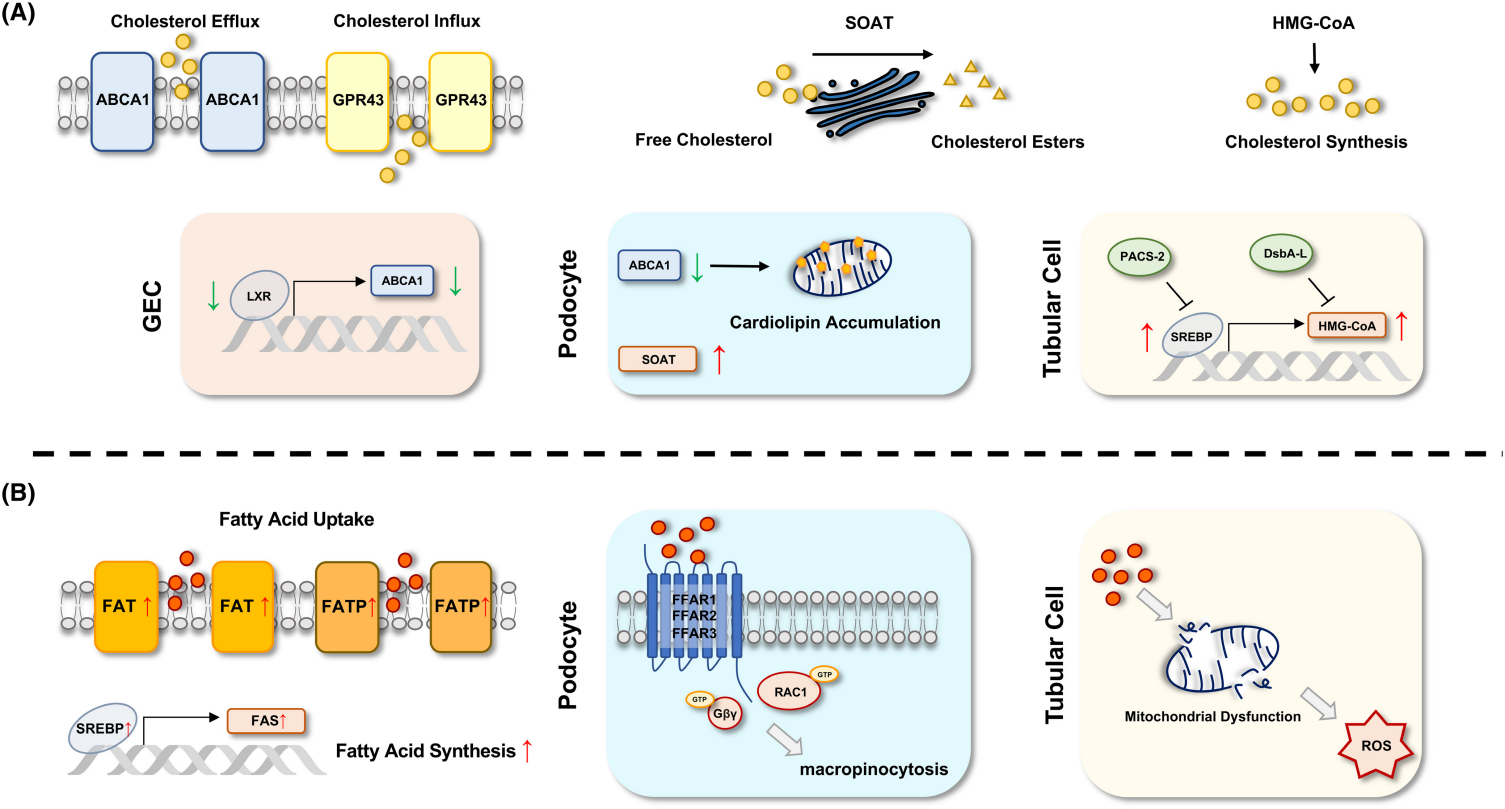
(A) Cholesterol in kidney resident cells is mainly regulated by cholesterol efflux regulatory gene ABCA1, cholesterol influx regulatory gene GPR43, cholesterol esterification regulatory gene SOAT and cholesterol synthesis rate‐limiting enzyme HMG‐CoA. Animal and in vitro studies have demonstrated that under diabetic conditions, the expression of ABCA1 is downregulated due to reduced LXR expression in GEC; in podocytes, reduction of ABCA1 leads to cardiolipin accumulation at mitochondrial, and SOAT expression correlates with intracellular cholesterol ester accumulation; in tubular cells, increase in SREBP results in elevated HMG‐CoA expression, thus accelerating cholesterol synthesis while PACS‐2 and DsbA‐L inhibits SREBP and HMG‐CoA, respectively. (B) In diabetic kidneys, cellular fatty acid uptake increases due to elevated expression of FAT and FATP. Besides, upregulated SREBP expression enhances fatty acid synthesis via FAS. In podocytes, the binding of free fatty acid and FFAR1/2/3 activates Gβ/Gγ complex and RAC1, triggers podocyte micropinocytosis; in tubular cells, free fatty acid overload generates mitochondrial dysfunction and ROS elevation. ABCA1, adenosine triphosphate‐binding cassette transporter A1; DsbA‐L, disulfide‐bond A oxidoreductase‐like protein; FAT, fatty acid translocase; FAS, fatty acid synthase; FATP, fatty acid transport proteins; FFAR, free fatty acid receptors; GEC, glomerular endothelial cell; GPR43, G‐protein‐coupled receptor 4; HMG‐CoA, β‐hydroxy β‐methylglutaryl‐CoA; LXR, liver X receptor; PACS‐2, phosphofurin acidic cluster sorting protein 2; ROS, reactive oxygen species; SOAT, sterol‐O‐acyltransferase; SREBP, sterol regulatory element‐binding protein.

In conclusion, it is widely acknowledged that lipid dysregulation and renal lipid accumulation contribute to DKD progression. Glomeruli and proximal tubule are the major kidney compartments affected by ectopic lipid accumulation. Current studies mainly focused on regulators in cholesterol and fatty acid metabolism in DKD kidneys. Genes involved in cholesterol and free fatty acid dysregulation in diabetic kidney resident cells were summarized in Table 1 and relevant pathways were illustrated in Figure 2. The role of oxysterol, a derivative of cholesterol, in DKD has been recently noticed. Targeting lipid accumulation remains challenging in DKD treatment. SGLT2 inhibitor, an FDA approved DKD therapy, seemed to prevent disease progression partially through regulating kidney lipid metabolism. Several potential therapeutic targets have also been identified and their inhibitors have shown renoprotective effects in DKD animal models; however, further validations are crucial before moving to clinical trials.

**TABLE 1:** Summary of genes involved in cholesterol and free fatty acid dysregulation in diabetic kidney resident cells.

Cell type	Cholesterol dysregulation		Free fatty acid dysregulation	
	Genes involved	Potential therapeutics	Genes involved	Potential therapeutics
Glomerular endothelial cell	LXR ABCA1	Exendin‐4	/	/
Podocyte	ABCA1 SOAT GPR43	ABCA1 inducer (A30) 5‐arylinicotinamide	FFAR FAT (CD36) JAML	PBI‐4050
Tubular cell	DsbA‐L PACS‐2 SOAT SREBPs	/	SLC27A2 KIM‐1 SREBPs	/

*Note*: Green and red indicates protective and harmful factors, respectively.

Abbreviations: ABCA1, adenosine triphosphate‐binding cassette transporter A1; DsbA‐L, disulfide‐bond A oxidoreductase‐like protein; FAT, fatty acid translocase; FFAR, free fatty acid receptors; GPR43, G‐protein‐coupled receptor 4; JAML, junctional adhesion molecule‐like protein; KIM‐1, kidney injury molecule‐1; LXR, liver X receptor; PACS‐2, phosphofurin acidic cluster sorting protein 2; SOAT, sterol‐O‐acyltransferase; SREBPs, sterol regulatory element‐binding proteins.

## AUTHOR CONTRIBUTIONS

**Zhengying Fang:** Writing – original draft (equal). **Ruijie Liu:** Writing – review and editing (supporting). **Jingyuan Xie:** Writing – review and editing (supporting). **John Cijiang He:** Funding acquisition (lead); writing – review and editing (lead).

## FUNDING INFORMATION

J.C.H. is supported by National Institute of Health (NIH)/National Institute of Diabetes and Digestive and Kidney Diseases (NIDDK) Grants: R01DK109683, R01DK122980, R01DK129467, R01DK56492 and VA Merit Award I01BX000345.

## CONFLICT OF INTEREST STATEMENT

The authors have no conflicts of interest to disclose.

## Data Availability

None.
